# Cross-sectional study of psychiatric disorders in patients with chronic musculoskeletal pain and individuals without pain

**DOI:** 10.1186/s42358-024-00375-x

**Published:** 2024-05-10

**Authors:** Ruben Horst Duque, Carla Vasconcelos Cáspar Andrade, Valdir Ribeiro Campos, Isac Ribeiro Moulaz, Laíssa Fiorotti Albertino, Maria Bernadete Renoldi de Oliveira Gavi

**Affiliations:** 1 Rheumatology Department, University Hospital Cassiano Antonio Moraes, Federal University of Espirito Santo, Vitoria, ES Brazil; 2 Neuromusculoskeletal Unit, University Hospital Cassiano Antonio Moraes, Federal University of Espirito Santo, Vitoria, ES Brazil; 3 Psychiatry Service, Internal Medicine Department, University Hospital Cassiano Antonio Moraes, Federal University of Espirito Santo, Vitoria, ES Brazil; 4 Federal University of Espirito Santo, Vitoria, ES Brazil

**Keywords:** Mental disorders, Musculoskeletal Pain, Chronic Pain, Fibromyalgia

## Abstract

**Background:**

Musculoskeletal chronic pain is a leading cause of global disability and laboral incapacity. However, there is a lack of population-based studies that investigate the relationship between chronic pain and mental disorders with a control group, particularly among low- and middle-income countries. Chronic pain is a serious public health problem in terms of human suffering, and in terms of socioeconomic implications. Frequent association with different mental disorders increases disability, decreases quality of life, and makes diagnosis and treatment challenging. The present study aimed to evaluate the presence of mental disorders in patients with chronic musculoskeletal pain and compare with a control group without pain.

**Methods:**

We selected 100 patients in a regular follow-up at the Musculoskeletal Pain Outpatient Clinic of the University Hospital and compared them with 100 painless individuals from the control group from June 2016 to June 2018. The instruments used were the Mini International Neuropsychiatric Interview (MINI-PLUS) and a structured questionnaire to collect sociodemographic data. Statistical analysis used t-test, chi-square, Fisher’s exact test, Mann-Whitney, Kolmogorov-Smirnov tests, and multiple logistic regression.

**Results:**

In the sample evaluated, the majority of patients were women (83%), of brown color (54%), with lower-level education (51%), lower salary range (73%) and high absenteeism rate at work (60,7%). Patients with chronic pain had more psychiatric disorders (88% vs. 48% in the control group; *p* < 0.001). The most frequent diagnoses were anxiety disorders with panic attacks (44%), generalized anxiety (36%), mixed anxiety and depression disorder (33%), social phobia (30%), agoraphobia (29%), suicide risk (28%), and major depression (27%).

**Conclusion:**

Positive correlations of mental disorders and chronic musculoskeletal pain have been documented. This suggests that psychiatric components must be taken into account in the management of chronic pain syndromes. The use of Mini Plus as a diagnostic tool for psychiatric disorders can contribute to optimizing the diagnosis and treatment of patients with chronic pain and encourage the creation of policies with strategies and criteria for quick access to Multi-professional Services.

**Supplementary Information:**

The online version contains supplementary material available at 10.1186/s42358-024-00375-x.

## Introduction

Chronic pain is a complex phenomenon that is pointed out by associated comorbidities. This topic has been an object of medical concern worldwide. In 2020, the International Association for the Study of Pain (IASP) approved a revised definition of pain with broad acceptance [1], which was embraced by the WHO (World Health Organization), as an unpleasant sensory and emotional experience associated with actual or potential tissue damage, or described in terms of such damage. In this scenario, the exact prevalence of musculoskeletal pain is hard to identify due to a wide prevalence range (10 to 50%) [2, 3] among studies. In Brazil, according to population studies, chronic pain is more prevalent among women; it is present in 41% of the population over 20 years old and in 51.4% of the population aged 60 and older [4, 5].

The association between chronic pain, social isolation, and psychiatric disorders is very common, such as sleep deprivation, anxiety, depression, financial issues, and, sometimes, suicide [6, 7]. It is estimated that 30–60% of patients with chronic pain present depressive symptoms [8–14]. There is evidence that central and cognitive influence in pain perception can be more important than the peripheral stimuli as exemplified by the analgesic effect of placebo [15]. However, the global association between chronic pain and mental disorders is still not clear, since it varies according clinical condition and individual susceptibility and involves mechanisms such as genetic influence, neurotransmitters interaction, central nervous system’s receptors, and pain-augmenting and pain-inhibiting neural circuits [16, 17]. The temporal relationship between chronic pain and mental alteration is still not fully understood, but it is probably bidirectional [18].

Few studies have identified risk factors that can be related with mental health issues in patients with chronic musculoskeletal pain. This study aimed to investigate the prevalence and the association between mental health disorders in patients with musculoskeletal chronic pain, compared to a control group, using a validated instrument for the identification of mental disorders and estimate risks of vulnerability in the population with chronic pain who were treated in a specialized Rheumatology Service.

## Materials and methods

This is a cross-sectional observational study. A total of 100 patients with chronic musculoskeletal pain were included randomly, after previous sorting by order of arrival in subsequent days in the Musculoskeletal Outpatient Pain Clinic of Rheumatology Service of Cassiano Antonio de Moraes Universitary Hospital (HUCAM) of Federal University of Espirito Santo, Vitoria-ES, Brazil (UFES) from June 2016 to June 2018. For the control group, after verbal invitation and signature of the Informed Consent Term to participate in the research, 100 individuals were selected from among the companions of patients in different outpatient clinics and wards (pediatrics, gynecology, medical clinic and surgical clinic), as well as some hospital employees.

Inclusion criteria were ages between 18 and 60 years old, ability to understand the questionnaires used during the interview, have a diagnosis of chronic musculoskeletal pain for more than 6 months (for the experimental group) or not presenting complaints of chronic pain, or rheumatological, orthopedic and neoplastic diseases (for the control group). Exclusion criteria were refusal to sign the consent form, visual and/or hearing impairment that compromised the use of instruments applied in the research.

### Instruments

#### Mini International Neuropsychiatric Interview (MINI-PLUS)

The MINI-PLUS is a questionnaire for the diagnosis of psychiatric disorders, derived from the “Composite International Diagnostic Interview” – CIDI [19, 20] – and adapted for the clinical context. The mood disorders evaluated by the MINI-PLUS are major depressive disorder, dysthymia, suicidal ideation and manic episodes. Among the anxiety disorders, the MINI-PLUS assesses panic disorder, agoraphobia, social phobia, obsessive-compulsive disorders, generalized anxiety disorder, and post-traumatic stress disorder.

The applicators of this research were oriented by an instructor trained by the author of the Brazilian version of the instrument and received authorization from the main author to use this instrument after paying a fee. This tool helps with diagnostic findings of the main mental disorders evaluated in the Diagnostic and Statistical Manual of Mental Disorder (DSM-V) [21, 22].

#### Sociodemographic questionnaire

A structured questionnaire was designed to collect sociodemographic data, as well as record information about the etiological diagnosis related to chronic pain, investigating the presence of associated clinical conditions.

### Data collection procedures

The data collection of the patients with chronic pain was carried out at the Musculoskeletal Pain Outpatient Clinic of the Rheumatology Service of University Hospital of UFES, by a trained evaluator; for the control group, data were collected according to the places where they were approached and invited to participate in the research, that is, wards and/or other clinics that are not related to pain. The participants answered a questionnaire, structured for the collection of sociodemographic data that included date of birth, color, race, weight, height, schooling, professional status, and clinical diagnosis referring to chronic pain. Finally, the MINI-PLUS questionnaire was applied in both groups by four well trained and capable clinical researchers. Two researchers were responsible for each group.

### Statistical analysis

Data analysis was performed using the IBM SPSS Statistics Program (Version 20). Categorical data are presented as the number of individuals and their respective percentages. Continuous variables are described as mean, median, and standard deviation. The normal distribution of the sample was verified using the Shapiro–Wilk and Kolmogorov-Smirnov tests, and the comparisons were made using Mann–Whitney’s non-parametric test and Chi-square test. The significance level was set at a *p*-value of < 0.05.

Univariate logistic regression was performed, analyzing the association of all independent variables (schooling, monthly family income, presence of psychiatric disorders) separately with the dependent variable under study (chronic pain). Subsequently, multiple logistic regression was performed, analyzing all the variables that presented potential for significance (*p*-value < 0.100) together, in order to verify what would actually be associated with the presence of the studied comorbidity. The measure of association calculated from the logistic model was the odds ratio (OR), considering the 95% confidence interval.

### Ethical aspects

This research has no public funding. All the necessary resources came from the researchers themselves. The research was approved by the Research Ethics Committee of the University Hospital on July 2, 2016.

## Results

The groups were homogeneous for sex, ethnicity, marital status and weight. There was a predominance of female, brown and married individuals in both groups. However, there were differences regarding age, income and absence from work – as shown below in Tables 1 and 2.

**Table 1:** Sociodemographic data - bivariate analysis

Variables	With pain	Without pain	*p*-value
**Sex [n (%)]**			0.849
Female	83 (83%)	84 (84%)	
**Race/Color [n (%)]**			0.098
White	35 (36.1%)	27 (27%)	
Brown	54 (55.7%)	55 (55%)	
Black	7 (7.2%)	12 (12%)	
Asian/Indigenous	1 (1%)	6 (6%)	
**Marital Status [n (%)]**			0.825
Single	19 (19.6%)	19 (19%)	
Married/Stable union	55 (56.7%)	61 (61%)	
Divorced	16 (16.5%)	12 (12%)	
Widower	7 (7.2%)	8 (8%)	
**Scholarity [n (%)]**			0.035
Illiterate	2 (2%)	3 (3%)	
Elementary school (complete or incomplete)	48 (49%)	39 (39%)	
Secondary school (complete or incomplete)	37 (37.8%)	31 (31%)	
Higher education	11 (11.2%)	27 (27%)	
**Away From Work [n (%)]**			< 0.001
No	35 (39.3%)	93 (93.9%)	
Yes	54 (60.7%)	6 (6.1%)	
**Professional status [n (%)]**			< 0.001
Unemployed / Student / Homemaker	41 (45.1%)	31 (31%)	
Retired / Sickness Allowance	8 (8.8%)	-	
Employed/ Working	42 (46.2%)	69 (69%)	
**Monthly Family Income [n (%)]**			< 0.001
< 1 Minimum Wage (MW)	8 (8.6%)	14 (14%)	
1 a 2 MW	60 (64.5%)	28 (28%)	
2 a 4 MW	18 (19.4%)	39 (39%)	
4 a 10 MW	7 (7.5%)	19 (19%)	
**Age – n (median [years])**	100 (50.5)	100 (41)	< 0.001**
**Weight – n (mean [Kg], SD)**	99 (73.28)	96 (70.78)	0.499
**Height – n (median [m])**	99 (1.60)	97 (1.65)	< 0.001**

** Mann-Whitney test

*p*-value: < 0.05

Patients in the pain group were older (49.1 ± 8.67 vs. 40.8 ± 10.87 years, *p* < 0.001) compared to the control group. Patients with pain had lower education (*p* = 0.035), and a lower frequency in higher education (11.2% vs. 27%) and elementary school (49% vs. 39%).

Moreover, 60.7% of people with chronic pain were away from work vs. only 6% in the control group (*p* < 0.001); only in the group with pain there were people receiving Sickness Allowance or retired (8.8% vs. 0%; *p* < 0.001). The average monthly income was between 1 and 2 minimum wages (MW) in the chronic pain group vs. 2 to 4 minimum wages (MW) in the control group (*p* < 0.001).

In the group of patients with chronic musculoskeletal pain, the main clinical conditions found were fibromyalgia (54%), mechanical low back pain (40%), mechanical neck pain (16%), tendinitis (7%), bursitis (5%), compressive syndromes (4%), myofascial pain (2%), thoracic pain (2%), and capsulitis (1%).

The presence of any mental disorder was found in 88% of the group with pain vs. 48% in the control group (*p* < 0.001). The detailed comparison between the prevalence of mental disorders in the experimental and control groups are shown below in Table 2.

**Table 2:** Characterization of mental disorders between groups

Variables	With pain	Without pain	*p*-value
	*n* (%)	*n* (%)	
**Current major depressive episode**			0.007
Yes	27(27%)	12 (12%)	
**Major Depressive Episode in the past**			0.462
Yes	16 (16%)	20 (20%)	
**Current Major Depressive Episode Due to General Medical Condition**			0.205
Yes	11 (11%)	6 (6%)	
**Major Depressive Episode Due to General Medical Condition in the past**			0.721*
Yes	3 (3%)	5 (5%)	
**Current Major Depressive Episode with Melancholic Features**			0.002
Yes	21 (21%)	6 (6%)	
**Current Major Depressive Episode with Melancholic Features in the past**			0.621*
Yes	3 (3%)	1 (1%)	
**Current Dysthymic Disorder**			0.004
Yes	21(21%)	7 (7%)	
**Dysthymic Disorder in the past**			0.246*
Yes	-	3 (3.0%)	
**Current suicide risk**			< 0.001
Yes	28 (28%)	7 (7%)	
**D.1 Current Manic Episode**			0.118*
Yes	6 (6%)	1 (1%)	
**D.2 Manic episode in the past**			0.059*
Yes	5 (5%)	-	
**Current Anxiety Disorder With Panic Attacks Due To General Medical Condition**			< 0.001
Yes	44 (44%)	8 (8%)	
**Lifetime Agoraphobia**			0.012
Yes	19 (19%)	7 (7%)	
**Current Agoraphobia** **Yes**	29(29%)	-	0.497*
**Current Panic Disorder without Agoraphobia**			0.497*
Yes	2 (2%)	-	
**Current Panic Disorder with Agoraphobia**			< 0.001
Yes	21 (21%)	1 (1%)	
**Current Social anxiety disorder**			< 0.001
Yes	30 (30%)	4 (4%)	
**Current Specific Phobia**			0.021
Yes	22 (22%)	10 (10%)	
**Current Obsessive Compulsive Disorder**			0.009
Yes	13 (13%)	3 (3%)	
**Current Psychotic Disorder Not Otherwise Specified**			0.017
Yes	10 (10%)	2 (2%)	
**Schizophrenia Criterion “A” Fulfilled Current**			0.721*
Yes	5 (5%)	3 (3%)	
**Current Generalized Anxiety Disorder**			< 0.001
Yes	36 (36%)	12 (12%)	
**Current Generalized Anxiety Disorder due to General Medical Condition**			0.621
Yes	10 (10%)	8 (8%)	
**Current Hypochondria**			< 0.001
Yes	15 (15%)	1 (1%)	
**Current Body Dysmorphic Disorder**			0.179
Yes	10 (10%)	5 (5%)	
**Pain disorder associated with psychological factors**			< 0.001
Yes	45 (45%)	2 (2%)	
**Adjustment Disorder**			0.721*
Yes	5 (5%)	3 (3%)	
**Current Probable Premenstrual Dysphoric Disorder**			0.060
Yes	4 (4%)	11 (11%)	
**Current Mixed Anxiety-Depression Disorder**			< 0.001
Yes	33 (33%)	9 (9%)	

* Fisher’s exact test

*** Test not performed due to null variables

The main anxiety disorders in patients with chronic pain were: anxiety disorder with panic attacks, generalized anxiety disorder, social phobia, agoraphobia, specific phobia, panic disorder with agoraphobia, post-traumatic stress disorder and obsessive-compulsive disorder. The most prevalent mood disorders were current major depressive episodes, depressive episodes with melancholic features and dysthymic disorder. Mixed anxiety and depression disorder was also found in 33% of patients with chronic pain.

In the control group, the most prevalent psychiatric disorders were generalized anxiety disorder and major depressive episode, both with a prevalence of 12%.

**Table 3:** Sociodemographic data analysis and psychiatric disorders – first part

Variables	Univariate analysis		
	*p*-value	OR	95%CI
**Age**	< 0.001	1.087	1.053–1.122
**Sex**			
Male	-	-	-
Female	0.849	0.930	0.441–1.963
**Race/Color**	0.132		
White	-	-	-
Brown/Black	0.256	0.702	0.382–1.293
Asian/Indigenous	0.065	0.129	0.015–1.133
**Marital Status**	0.826		
Single	-	-	-
Married/ Stable union	0.782	0.902	0.433–1.876
Divorced	0.566	1.333	0.499–3.560
Widower	0.827	0.875	0.264–2.897
**Scholarity**	0.047		
Illiterate	0.615	1.636	0.240–11.180
Elementary school (complete or incomplete)	0.008	3.021	1.332–6.849
Secondary school (complete or incomplete)	0.013	2.930	1.255–6.841
Higher education	-	-	-
**Away from work**			
No	-	-	-
Yes	< 0.001	23.914	9.448–60.531
**Professional Status**	0.042		
Unemployed / Student / Homemaker	0.012	2.173	1.188–3.975
Retired / Sickness Allowance	0.999	-	-
Employed/ Working	-	-	-
**Montly Family Income**	< 0.001		
Less than 1 MW	0.483	1.551	0.455–5.291
1 a 2 MW	< 0.001	5.816	2.192–15.432
2 a 4 MW	0.668	1.253	0.447–3.512
4 a 10 MW	-	-	-
**Psychiatric disorder**			
Yes	< 0.001	7.944	3.869–16.313
No	-	-	-

**Table 3:** Sociodemographic data analysis and psychiatric disorders – second part

Multivariate logistic regression analysis				
Variables	*p*-value	ORcrude	*p*-Value	ORajusted
**Age**	< 0.001	1.087 (1.053–1.122)	0.001	1.080 (1.031–1.131)
**Scholarity**				
Illiterate	0.615	1.636 (0.240–11.180)	0.315	0.246 (0.016–3.798)
Elementary school (complete or incomplete)	0.008	3.021 (1.332–6.849)	0.446	0.567 (0.131–2.443)
Secondary school (complete or incomplete)	0.013	2.930 (1.255–6.841)	0.837	0.861 (0.207–3.581)
Higher education	-	-	-	-
**Monthly Family Income**				
Less than 1 Minimum Wage (MW)	0.483	1.551 (0.455–5.291)	0.422	0.432 (0.056–3.351)
1 a 2 MW	< 0.001	5.816 (2.192–15.432)	0.398	1.991 (0.403–9.849)
2 a 4 MW	0.668	1.253 (0.447–3.512)	0.892	1.113 (0.238–5.201)
4 a 10 MW	-	-	-	-
**Psychiatric disorder**				
Yes	< 0.001	7.944 (3.869–16.313)	< 0.001	8.861 (3.066–25.607)
No	-	-	-	-

Statistical analysis using univariate logistic regression and multivariate logistic regression showed that factors such as age and having a psychiatric disorder were correlated with a greater chance of having chronic pain, with an adjusted OR of 1.080 (95%CI 1.031–1.131) and 8,861 (95%CI 3,066 − 25,607) respectively (Table 3).

## Discussion

This study was conducted in an outpatient service specialized in chronic musculoskeletal pain, aiming at a more efficient approach, within a multidisciplinary view, to identify mental disorders, and map the clinical profile of patients using an international validated standardized psychiatric questionnaire, the MINI-PLUS. The combined analysis of three international cross-sectional surveys showed that musculoskeletal pain has a negative impact on emotional well-being and quality of life, suggesting the need to adopt a biopsychosocial approach to pain that allows assessing not only pain intensity, but also the extent to which it negatively affects an individual’s life [23].

We identified that 88% of patients with chronic pain had some type of psychiatric disorder that had not been diagnosed until the assessment. The co-occurrence of two or more psychiatric disorders has been described as a common phenomenon [24], and it was also observed in the studied population. In this scenario, early multidisciplinary approaches can prevent tragic outcomes, as 28% of the studied population was at risk of suicide.

Socioeconomic disadvantages are associated with an increased risk of chronic pain. Individuals with low socio-educational level have inherent difficulties and less effective strategies to deal with pain. They have greater restrictions to access the health systems and less information about their condition, leading to a greater tendency for catastrophizing [25]. In addition, the adverse occupational factors, such as work activity with greater physical demand, limited autonomy, and low job satisfaction, can contribute to a worse prognosis. In population studies, the occurrence of chronic pain is inversely proportional to socioeconomic status, with evidence that people living in adverse circumstances experience more chronic pain and in greater severity independent of other clinical and demographic factors [26]. Our study demonstrated that the chronic pain group had a lower salary range (64.5% of patients earn between 1 and 2 minimum wages) and a higher rate of absence from work (60.7%) documented.

Regarding the prevalence of chronic pain, it is estimated that the national prevalence of chronic pain is around 41% of the population over 20 years old and 51.4% in people over 60 years old, being more frequent in women [5]. Moreover, elderly people are more likely to develop chronic and degenerative processes that lead to chronic pain and the comorbidities involved in this universe [27]. We observed similarities within the literature [4, 28], that also show that chronic pain is more prevalent among older people, and is more frequently correlated with females and with low socioeconomic and cultural status.

A multicenter population study in 15 centers, conducted in Asia, Africa, Europe and America, demonstrated differences in the prevalence of chronic pain influenced by several cultural and cross-cultural aspects, and that patients with chronic pain are more likely to have anxiety and depression disorders (OR 4.14; 95% CI 3.52–4.86) [29]. The mental disorder was an independent risk factor for chronic pain (OR = 8.86 95% CI 3.06–25.6) [12].

In this scenario, it is necessary to think about public health strategies that seek the prevention and early identification of these problems in order to establish a multidisciplinary approach that restores these patients’ dignity and quality of life, since, as constantly emphasized by the literature and corroborated by this work, chronic pain has no adaptive purpose and brings drastic consequences such as social isolation, psychological stress and psychiatric comorbidities [7]. We observed that 88% of the patients with chronic pain had some psychiatric comorbidity. Other studies are in line with the findings in this research [30, 31]. A large number of them did not have access to regular monitoring by a mental health professional, much less the diagnosis of psychiatric comorbidity. Furthermore, in this post-pandemic period, social isolation contributes to the worsening of chronic health conditions, especially psychiatric disorders [32].

Regarding the assessment of mental disorders, our study used the MINI-PLUS as a diagnostic tool, which is a structured interview, lasting from 15 to 30 min, and compatible with the diagnostic criteria used in clinical practice. This is an inexpensive strategy, easily applied by clinicians after training from 1 to 3 h. The instrument demonstrates high sensitivity and also specificity, with the ability to exclude people without disorders [21]. The literature records the use of this tool in several clinical profiles such as ischemic stroke, chronic lung disease (COPD), liver transplantation and rheumatological diseases. In an extensive review of the literature on MINI-PLUS in rheumatic diseases, we found only two articles where the authors used this questionnaire to study patients with Systemic Lupus Erythematosus [32–34]. Our study did not include autoimmune diseases.

Similar to the literature, the most prevalent mood disorders found in the studied patients were: major depression (27%), major depression with melancholic features (21%), dysthymic disorder (21%), and current suicide risk (28%) [35]. Van Korff et al, in a population study conducted in the United States, surveying 5692 community residents and Demyttenaere et al, in a transnational study with more than 85,000 people from communities in 18 countries evaluated chronic cervical pain, lumbar pain, anxiety and mood disorders [36, 37]. Both studies used the third version of the “Composite International Diagnostic Interview’’ (CIDI). In the first study, a prevalence of mood disorders of 17.5% was described, with major depression being the most prevalent disorder (12.6%). In the multicenter study, the prevalence of depression ranged from 2.5–15%. Similarly, the “STOP-PAIN” project, a cross-sectional study in Canada that evaluated 728 people waiting for treatment at a multidisciplinary pain center, described 82% of the presence of depressive symptoms (using the “Beck Depression inventory”) with 34.6% reporting suicidal ideation [38]. The data from the present study also showed a higher prevalence of current suicide risk in patients with chronic pain compared to the control group (28% vs 7%, respectively).

Like other specialized centers, fibromyalgia was the most common clinical condition evaluated. Fibromyalgia is among the most frequent causes of chronic musculoskeletal pain, associated with longer pain and seeking of medical resources [5]. It is probably the most important central sensitization pain syndrome in which an abnormal response of the central nervous system to peripheral stimuli due to hypersensitivity and hyperexcitability is described, characterizing prolonged or persistent pain [39], leading to a close correlation with psychiatric disorders, mainly depression and anxiety [40], but also a 10.5 times greater risk of suicide than the general population [41].

In a case-control study with 8,879 patients with fibromyalgia, 34 suicide attempts were described and 96 cases of suicidal ideation were documented. The risk factors for suicide identified were: fatigue (OR = 1.29, 95% CI 1.25–1.32), dizziness (OR = 1.25, 95% CI 1.22–1.28), weakness (OR = 1.17, 95% CI 1.07–1.18), obesity with BMI 50–59 (OR = 1.18 95% CI 1.12–1.18), drug dependence (OR = 1.18 95% CI 1.1–1.27), and mental disorder – depression/psychosis – (OR 1.12 95% CI 1.07–1.18). In contrast, regularity in outpatient clinical follow-up with prescription adjustments for both the general medical condition and the psychological condition are protective factors [41].

In general, chronic pain correlated with a greater propensity for suicidal thoughts and behavior [40, 42, 43] which includes from migraine (OR 4.4) and non-migraine headache (OR = 6.2) to psychogenic pain [44, 45].

Concerning anxiety disorders, we observed that 44% of patients had a current anxiety disorder with panic attacks due to general medical condition, 29% had agoraphobia, and 21% had associated panic disorder, 30% with social phobia, 15% with hypochondria, 17% with post-traumatic stress disorder, 36% with generalized anxiety and 33% with mixed anxiety disorder and current depression. Van Korff et al. described an OR = 1.5 (95% CI 0.9–2.4) for agoraphobia in patients with chronic low back pain, and an OR = 2.6 (95% CI 2.1–3.3) for post-traumatic stress disorder. Pooled OR for any anxiety disorder was 2.3 (95% CI 1.9–2.7) [36].

In a multicenter study of patients with chronic low back pain and neck pain, social phobia presented OR 1.9 (95% CI 1.7–2.2) and generalized anxiety disorder OR 2.7 (95% CI 2.4–3.1). Also in this study, the OR pooled for any anxiety disorder was 2.2 (95% CI 2.1–2.4) [37]. Unlike the aforementioned studies, which used the third version of the CIDI, we used the MINI-PLUS and, similarly, anxiety disorders were very prevalent. Fishbain et al., in a structured evidence-based review, supported an association between hypochondria and chronic pain [44, 45]. We found 15% of hypochondriac patients in our sample.

Post-Traumatic Stress Disorder was observed in 17% of our chronic pain patients. There is a growing body of research demonstrating that chronic pain is more common among people who report a history of abuse and/or violence at any age, both in domestic and public settings [28, 46]. This data can be correlated with the socioeconomic and cultural reality of the studied population, but it was not the objective of this study. Another impact factor was the frequent association of pain, depression and anxiety (33% in our sample). These associations may contribute to a worse prognosis according to the literature, which reports that the three factors have independent and additive adverse effects on the intensity and interference of pain, functional limitations and time of disability [13]. Both in clinical samples and in population studies, the occurrence of two or more mental disorders is a common phenomenon. Approximately 30% of patients with a psychiatric disorder in the 12 months prior to the interview had two or more associated disorders [24].

We found in the literature only two studies that compared a group of patients with chronic pain with a control group without pain. In these studies, chronic pain diagnoses were not specified and the Mini Plus questionnaire was not used [47, 48].

Finally, it is worth noting that this study had limitations, as it is an observational study and also because of the number of patients recruited. The recruitment of individuals from different wards and outpatient clinics consists in a selection bias that we mitigate with randomization and a control group. Despite these limitations, the results found, in general, were not discrepant from those observed in the literature and do not invalidate the issues already discussed above.

## Conclusion

Psychiatric disorders are under diagnosed in patients with chronic musculoskeletal pain. This information should be considered in the management of these patients.

The use of the MINI-PLUS questionnaire showed effectiveness in identifying, among the evaluated patients with chronic pain, that 88% of them had some psychiatric disorder. The most prevalent disorders observed were: anxiety disorder with panic attacks, generalized anxiety disorder, mixed depression and anxiety disorder, social phobia, agoraphobia, suicide risk and major depressive disorder.

This study suggests an important need for integration of rheumatology and mental health services and generated hypotheses for future research for a better understanding of both chronic pain and mental disorders. The use of the diagnostic tool used in this study can facilitate and speed up the identification of psychiatric disorders by clinicians in patients with chronic pain, contribute to a more appropriate approach and reduce treatment costs.

## Electronic supplementary material

Below is the link to the electronic supplementary material.

Supplementary Material 1

Supplementary Material 2

Supplementary Material 3

## Data Availability

All data generated or analysed during this study are included in this published article [and its supplementary information files].
