# Supplementary material for: Cross-sectional study of psychiatric disorders in patients with chronic musculoskeletal pain and individuals without pain
Source: Adv Rheumatol. 2024 May 10;64:40. doi: 10.1186/s42358-024-00375-x (PMC13488751; doi:10.1186/s42358-024-00375-x)
Supplement: Supplementary file 1 [file 42358_2024_375_MOESM1_ESM.pdf]

CERTIFICADO

Ministério da Educação
Universidade Federal de Minas Gerais
Pró-Reitoria de Extensão
ATUALIZAÇÃO

CERTIFICAMOS que ***VALDIR RIBEIRO CAMPOS***

participou do ***TREINAMENTO NO USO DO "MINI INTERNATIONAL NEUROPSYCHIATRIC INTERVIEW" ministrado por PATRÍCIA AMORIM,***

promovido pelo Departamento de Saúde Mental da Faculdade de Medicina. Apoio: Associação Acadêmica Psiquiátrica de Minas Gerais – AAP-MG (Federada da Associação Brasileira de Psiquiatria – ABP) e Centro de Extensão da Faculdade de Medicina.

Realizado no dia **30 de agosto de 2003**, com a carga horária de **08 horas.**

Belo Horizonte, 10 de outubro de 2003.

Prof. Maurício Viotti Daker
Coordenador do Curso

Drª. Gislene Valadares Miranda
Subcoordenadora do Curso

Prof. Ari de Pinho Tavares
Coordenador do Centro de Extensão

Prof. Geraldo Brasileiro Filho
Diretor da Faculdade de Medicina

Comp4SÁBADOSDEFORMAÇÃOCmini

CENTRO DE EXTENSÃO DA F. M. UFMG
Acha-se o presente certificado registrado
sob o n.º MED. 03-02/79 32
no livro 04, folha 07
Belo Horizonte, 10 / 10 / 03
SECRETARIA DO CENEX - F. M. UFMG
