# Supplementary material for: Cross-sectional study of psychiatric disorders in patients with chronic musculoskeletal pain and individuals without pain
Source: Adv Rheumatol. 2024 May 10;64:40. doi: 10.1186/s42358-024-00375-x (PMC13488751; doi:10.1186/s42358-024-00375-x)
Supplement: Supplementary file 2 [file 42358_2024_375_MOESM3_ESM.pdf]

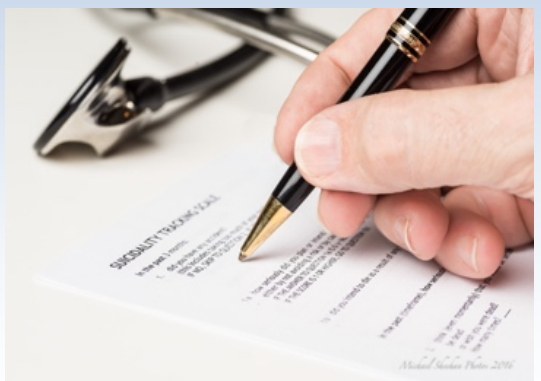

INVOICE

# PAID ON 3/1/2024 on Paypal


David V Sheehan MD MBA

Author and copyright holder of structured diagnostic interviews and rating scales for measurement-based healthcare

611 Warren Rd, Lutz, FL - 33548
Phone: 813.956.8437
Email: davidvsheehan@gmail.com


INVOICE # 20240228.1
DATE: FEBRUARY 28, 2024

SOLD TO
Valdir Ribeiro Campos
Federal University of Espirito
Santo Rheumatology Department, Santo, 1355, Marechal
Campos Avenue, Vitoria-ES, Brazil 29047-105.
☎Phone: +55 27 33357201
Email: vrcampos@terra.com.br

PAID

| PAYMENT METHOD | DAVID SHEEHAN SOCIAL SECURITY (TAX ID) NUMBER | PAYMENT TERMS | PO NUMBER |
|---|---|---|---|
| Check / Square / PayPal / Bank Wire Transfer / ACH | 044-54-2837 | On Receipt | |

| QTY | ITEM # | DESCRIPTION | UNIT PRICE | CREDIT | LINE TOTAL ($ USD) |
|---|---|---|---|---|---|
| 200 | | License fee for use of the MINI 5.0 in Federal University of Espirito Santo @plataformabrasil.saude.gov.br # 031955/20[illegible] IRB # 55249116.5.0000.[illegible]71 only<br><br>Please send check payment to:<br>David V Sheehan, MD, MBA<br>611 Warren Rd,<br>Lutz, FL, USA 33548<br><br>You can use my davidvsheehan@gmail.com to send the PayPal payment.<br><br>To pay via ACH or wire transfer, use:<br>Bank Name: JP Morgan Chase<br>Bank address: 16534 N Florida Ave, Lutz, FL 33549<br>Account Name: David V Sheehan<br>Account Number: 896332126<br>Routing Number (ABA/FW number): 267084131<br>IBAN Number: US Banks do not use an IBAN Number<br>Swift Code: CHASUS33<br><br>Please reference the Invoice number above with the payment for ease of tracking. | $15.00 | | $3,000.00 |
| | | | TOTAL | | $3,000.00 |

| Service Fees | USA | OUS |
|---|---|---|
| Paypal | 3% | 5% |
| Square | 3% | 3% |
| ACH | 0 | 0 |
| Bank Wire | $25 | $50 |
| Check | 0 | 0 |

*Tax Withholding will require an additional service fee of 10% for additional workload to document for your country
