# Supplementary material for: Cross-sectional study of psychiatric disorders in patients with chronic musculoskeletal pain and individuals without pain
Source: Adv Rheumatol. 2024 May 10;64:40. doi: 10.1186/s42358-024-00375-x (PMC13488751; doi:10.1186/s42358-024-00375-x)
Supplement: Supplementary file 3 [file 42358_2024_375_MOESM2_ESM.docx]

**Table 2 – Characterization of mental disorders between groups**

| **Variables** | **With pain** | | | | | | | | | | | | **Without pain** | | | | | | | | | | | | **p-value** | | | | | |  |  |  |  |  |
| --- | --- | --- | --- | --- | --- | --- | --- | --- | --- | --- | --- | --- | --- | --- | --- | --- | --- | --- | --- | --- | --- | --- | --- | --- | --- | --- | --- | --- | --- | --- | --- | --- | --- | --- | --- |
|  | **n (%)** | | | | | | | | | | | | **n (%)** | | | | | | | | | | | |  |  |  |  |  |  |  |  |  |  |  |
| **A.1 - Current major depressive episode** |  | | | | | |  | | | | | |  | | | | | |  | | | | | | 0.007 | | | | | |  |  |  |  |  |
| Yes | 27(27%) | | | | | | | | | | | | 12 (12%) | | | | | | | | | | | |  |  |  |  |  |  |  |  |  |  |  |
| **A.2 - Major Depressive Episode in the past** |  | | | | | |  | | | | | |  | | | | | |  | | | | | | 0.462 | | | | | |  |  |  |  |  |
| Yes | 16 (16%) | | | | | | | | | | | | 20 (20%) | | | | | | | | | | | |  |  |  |  |  |  |  |  |  |  |  |
| **A.3 - Current Major Depressive Episode Due to General Medical Condition** |  | | | | | |  | | | | | |  | | | | | |  | | | | | | 0.205 | | | | | |  |  |  |  |  |
| Yes | 11 (11%) | | | | | | | | | | | | 6 (6%) | | | | | | | | | | | |  |  |  |  |  |  |  |  |  |  |  |
| **A.4 - Major Depressive Episode Due to General Medical Condition in the past** |  | | | | | |  | | | | | |  | | | | | |  | | | | | | 0.721* | | | | | |  |  |  |  |  |
| Yes | 3 (3%) | | | | | | | | | | | | 5 (5%) | | | | | | | | | | | |  |  |  |  |  |  |  |  |  |  |  |
| **A.5 - Current Substance-induced Major Depressive episode** |  | | | | | |  | | | | | |  | | | | | |  | | | | | | *** | | | | | |  |  |  |  |  |
| Yes | - | | | | | | | | | | | | - | | | | | | | | | | | |  |  |  |  |  |  |  |  |  |  |  |
| **A.6 - Substance-induced Major Depressive episode in the past** |  | | | | | |  | | | | | |  | | | | | |  | | | | | | 0.497* | | | | | |  |  |  |  |  |
| Yes | 2 (2%) | | | | | | | | | | | | - | | | | | | | | | | | |  |  |  |  |  |  |  |  |  |  |  |
| **A.7 - Current Major Depressive Episode with Melancholic Features** |  | | | | | |  | | | | | |  | | | | | |  | | | | | | 0.002 | | | | | |  |  |  |  |  |
| Yes | 21 (21%) | | | | | | | | | | | | 6 (6%) | | | | | | | | | | | |  |  |  |  |  |  |  |  |  |  |  |
| **A.8 - Current Major Depressive Episode with Melancholic Features in the past** |  | | | | | |  | | | | | |  | | | | | |  | | | | | | 0.621* | | | | | |  |  |  |  |  |
| Yes | 3 (3%) | | | | | | | | | | | | 1 (1%) | | | | | | | | | | | |  |  |  |  |  |  |  |  |  |  |  |
| **B.1 - Current Dysthymic Disorder** |  | | | | | |  | | | | | |  | | | | | |  | | | | | | 0.004 | | | | | |  |  |  |  |  |
| Yes | 21(21%) | | | | | | | | | | | | 7 (7%) | | | | | | | | | | | |  |  |  |  |  |  |  |  |  |  |  |
| **B.2 - Dysthymic Disorder in the past** |  | | | | | |  | | | | | |  | | | | | |  | | | | | | 0.246* | | | | | |  |  |  |  |  |
| Yes | - | | | | | | | | | | | | 3 (3.0%) | | | | | | | | | | | |  |  |  |  |  |  |  |  |  |  |  |
| **C - Current suicide risk** |  | | | | | |  | | | | | |  | | | | | |  | | | | | | <0.001 | | | | | |  |  |  |  |  |
| Yes | 28 (28%) | | | | | | | | | | | | 7 (7%) | | | | | | | | | | | |  |  |  |  |  |  |  |  |  |  |  |
| **D.1 Current Hypomanic Episode** | | | |  | | | |  | | | | | |  | | | | | |  | | | | | | 1.000* | | | | | |  |  |  |  |
| Yes | | | | - | | | | | | | | | | 1 (1%) | | | | | | | | | | | |  |  |  |  |  |  |  |  |  |  |
| **D.2 Hypomanic Episode in the past** | | | |  | | | |  | | | | | |  | | | | | |  | | | | | | 0.246* | | | | | |  |  |  |  |
| Yes | | | | 3 (3%) | | | | | | | | | | - | | | | | | | | | | | |  |  |  |  |  |  |  |  |  |  |
| **D.3 Current Manic Episode** | | | |  | | | |  | | | | | |  | | | | | |  | | | | | | 0.118* | | | | | |  |  |  |  |
| Yes | | | | 6 (6%) | | | | | | | | | | 1 (1%) | | | | | | | | | | | |  |  |  |  |  |  |  |  |  |  |
| **D.4 Manic episode in the past** | | | |  | | | |  | | | | | |  | | | | | |  | | | | | | 0.059* | | | | | |  |  |  |  |
| Yes | | | | 5 (5%) | | | | | | | | | | - | | | | | | | | | | | |  |  |  |  |  |  |  |  |  |  |
| **D.5- Current (Hypo) Manic Episode Due to General Medical Condition** | | | |  | | | |  | | | | | |  | | | | | |  | | | | | | 1.000* | | | | | |  |  |  |  |
| Yes | | | | 1 (1%) | | | | | | | | | | - | | | | | | | | | | | |  |  |  |  |  |  |  |  |  |  |
| **D.6- (Hypo) Manic Episode Due to General Medical Condition in the past** | | | |  | | | |  | | | | | |  | | | | | |  | | | | | | 1.000* | | | | | |  |  |  |  |
| Yes | | | | 2 (2%) | | | | | | | | | | 1 (1%) | | | | | | | | | | | |  |  |  |  |  |  |  |  |  |  |
| **D.7 - Current substance-induced (Hypo) Manic Episode** | | | |  | | | |  | | | | | |  | | | | | |  | | | | | | 1.0000* | | | | | |  |  |  |  |
| Yes | | | | 1 (1%) | | | | | | | | | | - | | | | | | | | | | | |  |  |  |  |  |  |  |  |  |  |
| **D.8 - Substance-induced (Hypo) Manic Episode in the past** | | | |  | | | |  | | | | | |  | | | | | |  | | | | | | *** | | | | | |  |  |  |  |
| Yes | | | | - | | | | | | | | | | - | | | | | | | | | | | |  |  |  |  |  |  |  |  |  |  |
| **D.9 - Rapid Cycling Bipolar Disorder** | | | |  | | | |  | | | | | |  | | | | | |  | | | | | | *** | | | | | |  |  |  |  |
| Yes | | | | - | | | | | | | | | | - | | | | | | | | | | | |  |  |  |  |  |  |  |  |  |  |
| **D.10 - Mixed episode** | | | |  | | | |  | | | | | |  | | | | | |  | | | | | | *** | | | | | |  |  |  |  |
| Yes | | | | - | | | | | | | | | | - | | | | | | | | | | | |  |  |  |  |  |  |  |  |  |  |
| **D.11 - Seasonal affective disorder** | | | |  | | | |  | | | | | |  | | | | | |  | | | | | | *** | | | | | |  |  |  |  |
| Yes | | | | - | | | | | | | | | | - | | | | | | | | | | | |  |  |  |  |  |  |  |  |  |  |
| **D.12 - With Complete Remission Between Episodes** | | | |  | | | |  | | | | | |  | | | | | |  | | | | | | *** | | | | | |  |  |  |  |
| Yes | | | | - | | | | | | | | | | - | | | | | | | | | | | |  |  |  |  |  |  |  |  |  |  |
| **E.1 - Current Anxiety Disorder With Panic Attacks Due To General Medical Condition** | |  | | | | | | |  | | | | | |  | | | | | |  | | | | | | <0.001 | | | | | |  |  |  |
| Yes | | 44 (44%) | | | | | | | | | | | | | 8 (8%) | | | | | | | | | | | |  |  |  |  |  |  |  |  |  |
| **E.2 - Current Substance-induced Anxiety Disorder With Panic Attacks** | |  | | | | | | |  | | | | | |  | | | | | |  | | | | | | *** | | | | | |  |  |  |
| Yes | | - | | | | | | | | | | | | | - | | | | | | | | | | | |  |  |  |  |  |  |  |  |  |
| **F.1 - Lifetime Agoraphobia** | |  | | | | | | |  | | | | | |  | | | | | |  | | | | | | 0.012 | | | | | |  |  |  |
| Yes | | 19 (19 %) | | | | | | | | | | | | | 7 (7%) | | | | | | | | | | | |  |  |  |  |  |  |  |  |  |
| **F.2 - Current Agoraphobia** | |  | | | | | | |  | | | | | |  | | | | | |  | | | | | | <0.001 | | | | | |  |  |  |
| Yes | | 29 (29%) | | | | | | | | | | | | | 5 (5%) | | | | | | | | | | | |  |  |  |  |  |  |  |  |  |
| **F.3 - Current Panic Disorder without Agoraphobia** | |  | | | | | | |  | | | | | |  | | | | | |  | | | | | | 0.497* | | | | | |  |  |  |
| Yes | | 2 (2%) | | | | | | | | | | | | | - | | | | | | | | | | | |  |  |  |  |  |  |  |  |  |
| **F.4 - Current Panic Disorder with Agoraphobia** | |  | | | | | | |  | | | | | |  | | | | | |  | | | | | | <0.001 | | | | | |  |  |  |
| Yes | | 21 (21%) | | | | | | | | | | | | | 1 (1%) | | | | | | | | | | | |  |  |  |  |  |  |  |  |  |
| **F.5 - Current Agoraphobia Atual Without history of Panic Disorder** | |  | | | | | | |  | | | | | |  | | | | | |  | | | | | | 1.000 | | | | | |  |  |  |
| Yes | | 1 (1%) | | | | | | | | | | | | | - | | | | | | | | | | | |  |  |  |  |  |  |  |  |  |
| **F.6 - Current Agoraphobia, without current Panic Disorder, but with history of Panic Disorder** | |  | | | | | | |  | | | | | |  | | | | | |  | | | | | | *** | | | | | |  |  |  |
| Yes | | - | | | | | | | | | | | | | - | | | | | | | | | | | |  |  |  |  |  |  |  |  |  |
| **G - Current Social anxiety disorder** | |  | | | | | | |  | | | | | |  | | | | | |  | | | | | | <0.001 | | | | | |  |  |  |
| Yes | | 30 (30%) | | | | | | | | | | | | | 4 (4%) | | | | | | | | | | | |  |  |  |  |  |  |  |  |  |
| **H - Current Specific Phobia** | |  | | | | | | |  | | | | | |  | | | | | |  | | | | | | 0.021 | | | | | |  |  |  |
| Yes | | 22 (22%) | | | | | | | | | | | | | 10 (10%) | | | | | | | | | | | |  |  |  |  |  |  |  |  |  |
| **I.1 - Current Obsessive Compulsive Disorder** | |  | | | | | | |  | | | | | |  | | | | | |  | | | | | | 0.009 | | | | | |  |  |  |
| Yes | | 13 (13%) | | | | | | | | | | | | | 3 (3%) | | | | | | | | | | | |  |  |  |  |  |  |  |  |  |
| **I.2 - Current Obsessive Compulsive Disorder Due To General Medical Condition** | | |  | | | | | | |  | | | | | |  | | | | | |  | | | | | | 1.000* | | | | | |  |  |
| Yes | | | 2 (2%) | | | | | | | | | | | | | 1 (1%) | | | | | | | | | | | |  |  |  |  |  |  |  |  |
| **I.3 - Current substance-induced Obsessive Compulsive Disorder** | | |  | | | | | | |  | | | | | |  | | | | | |  | | | | | | *** | | | | | |  |  |
| Yes | | | - | | | | | | | | | | | | | - | | | | | | | | | | | |  |  |  |  |  |  |  |  |
| **J – Current Post Traumatic Stress Disorder** | | |  | | | | | | |  | | | | | |  | | | | | |  | | | | | | 0,000 | | | | | |  |  |
| Yes | | | 17 (17%) | | | | | | | | | | | | | 2 (2%) | | | | | | | | | | | |  |  |  |  |  |  |  |  |
| **K.1 – Current alcohol dependence disorder** | | |  | | | | | | |  | | | | | |  | | | | | |  | | | | | | 0.246* | | | | | |  |  |
| Yes | | | - | | | | | | | | | | | | | 3 (3%) | | | | | | | | | | | |  |  |  |  |  |  |  |  |
| **K.2 – Current alcohol abuse disorder** | | |  | | | | | | |  | | | | | |  | | | | | |  | | | | | | 0.497* | | | | | |  |  |
| Yes | | | - | | | | | | | | | | | | | 2 (2%) | | | | | | | | | | | |  |  |  |  |  |  |  |  |
| **K.3 – Lifetime alcohol dependence disorder** | | |  | | | | | | |  | | | | | |  | | | | | |  | | | | | | 1.000* | | | | | |  |  |
| Yes | | | - | | | | | | | | | | | | | 1 (1%) | | | | | | | | | | | |  |  |  |  |  |  |  |  |
| **K.4 – Lifetime alcohol abuse disorder** | | |  | | | | | | |  | | | | | |  | | | | | |  | | | | | | 1.000* | | | | | |  |  |
| Yes | | | - | | | | | | | | | | | | | 1 (1%) | | | | | | | | | | | |  |  |  |  |  |  |  |  |
| **L.1 - Lifetime Substance(S) Addiction** | | |  | | | | | | |  | | | | | |  | | | | | |  | | | | | | *** | | | | | |  |  |
| Yes | | | - | | | | | | | | | | | | | - | | | | | | | | | | | |  |  |  |  |  |  |  |  |
| **L.2 – Current Substance (S) addiction** | | |  | | | | | | |  | | | | | |  | | | | | |  | | | | | | *** | | | | | |  |  |
| Yes | | | - | | | | | | | | | | | | | - | | | | | | | | | | | |  |  |  |  |  |  |  |  |
| **L.3 - Current Substance (S) abuse** | | |  | | | | | | |  | | | | | |  | | | | | |  | | | | | | *** | | | | | |  |  |
| Yes | | | - | | | | | | | | | | | | | - | | | | | | | | | | | |  |  |  |  |  |  |  |  |
| **M.1 -Current Psychotic Disorder Not Otherwise Specified** | | |  | | | | | | |  | | | | | |  | | | | | |  | | | | | | 0.017 | | | | | |  |  |
| Yes | | | 10 (10%) | | | | | | | | | | | | | 2 (2%) | | | | | | | | | | | |  |  |  |  |  |  |  |  |
| **M.2 -Lifetime Psychotic Disorder Not Otherwise Specified** | | | | |  | | | | | |  | | | | | |  | | | | | |  | | | | | | 0.246* | | | | | |  |
| Yes | | | | | 3 (3%) | | | | | | | | | | | | - | | | | | | | | | | | |  |  |  |  |  |  |  |
| **M.3 - Schizophrenia Criterion "A" Not Fulfilled Current** | | | | |  | | | | | |  | | | | | |  | | | | | |  | | | | | | 1.000* | | | | | |  |
| Yes | | | | | - | | | | | | | | | | | | 1 (1%) | | | | | | | | | | | |  |  |  |  |  |  |  |
| **M.4 - Schizophrenia Criterion "A" Fulfilled Current** | | | | |  | | | | | |  | | | | | |  | | | | | |  | | | | | | 0.721* | | | | | |  |
| Yes | | | | | 5 (5%) | | | | | | | | | | | | 3 (3%) | | | | | | | | | | | |  |  |  |  |  |  |  |
| **N- Current Anorexia Nervosa** | | | | |  | | | | | |  | | | | | |  | | | | | |  | | | | | | *** | | | | | |  |
| Yes | | | | |  | | | | | | | | | | | |  | | | | | | | | | | | |  |  |  |  |  |  |  |
| **O.1 – Current Bulimia Nervosa** | | | | |  | | | | | |  | | | | | |  | | | | | |  | | | | | | 1.000* | | | | | |  |
| Yes | | | | | - | | | | | | | | | | | | 1 (1%) | | | | | | | | | | | |  |  |  |  |  |  |  |
| **O.2 - Bulimia Nervosa Purging Type** | | | | |  | | | | | |  | | | | | |  | | | | | |  | | | | | | 1.000* | | | | | |  |
| Yes | | | | | - | | | | | | | | | | | | 1 (1%) | | | | | | | | | | | |  |  |  |  |  |  |  |
| **O.3 - Anorexia Nervosa Binge-Binging/Purging Current Type** | | | | |  | | | | | |  | | | | | |  | | | | | |  | | | | | | *** | | | | | |  |
| Yes | | | | | - | | | | | | | | | | | | - | | | | | | | | | | | |  |  |  |  |  |  |  |
| **O.4 - Anorexia Nervosa Current Restrictive Type** | | | | |  | | | | | |  | | | | | |  | | | | | |  | | | | | | *** | | | | | |  |
| Yes | | | | | - | | | | | | | | | | | | - | | | | | | | | | | | |  |  |  |  |  |  |  |
| **P.1 - Current Generalized Anxiety Disorder** | | | | |  | | | | | |  | | | | | |  | | | | | |  | | | | | | <0.001 | | | | | |  |
| Yes | | | | | 36 (36%) | | | | | | | | | | | | 12 (12%) | | | | | | | | | | | |  |  |  |  |  |  |  |
| **P.2 - Current Generalized Anxiety Disorder due to General Medical Condition** | | | | |  | | | | | |  | | | | | |  | | | | | |  | | | | | | 0.621 | | | | | |  |
| Yes | | | | | 10 (10%) | | | | | | | | | | | | 8 (8%) | | | | | | | | | | | |  |  |  |  |  |  |  |
| **P.3 – Current Substance-induced Generalized Anxiety Disorder** | | | | |  | | | | | |  | | | | | |  | | | | | |  | | | | | | 1.000* | | | | | |  |
| Yes | | | | | 2 (2%) | | | | | | | | | | | | 1 (1%) | | | | | | | | | | | |  |  |  |  |  |  |  |
| **Q - Lifetime Antisocial Personality Disorder** | | | | |  | | | | | |  | | | | | |  | | | | | |  | | | | | | *** | | | | | |  |
| Yes | | | | | - | | | | | | | | | | | | - | | | | | | | | | | | |  |  |  |  |  |  |  |
| **R.1 - Lifetime Somatization Disorder** | | | | | |  | | | | | |  | | | | | |  | | | | | |  | | | | | | *** | | | | | |
| Yes | | | | | | - | | | | | | | | | | | | - | | | | | | | | | | | |  |  |  |  |  |  |
| **R.2 - Current Somatization Disorder** | | | | | |  | | | | | |  | | | | | |  | | | | | |  | | | | | | *** | | | | | |
| Yes | | | | | | - | | | | | | | | | | | | - | | | | | | | | | | | |  |  |  |  |  |  |
| **S – Current Hypochondria** | | | | | |  | | | | | |  | | | | | |  | | | | | |  | | | | | | <0.001 | | | | | |
| Yes | | | | | | 15 (15%) | | | | | | | | | | | | 1 (1%) | | | | | | | | | | | |  |  |  |  |  |  |
| **T - Current Body Dysmorphic Disorder** | | | | | |  | | | | | |  | | | | | |  | | | | | |  | | | | | | 0.179 | | | | | |
| Yes | | | | | | 10 (10%) | | | | | | | | | | | | 5 (5%) | | | | | | | | | | | |  |  |  |  |  |  |
| **U.1 - Pain disorder associated with psychological factors** | | | | | |  | | | | | |  | | | | | |  | | | | | |  | | | | | | <0.001 | | | | | |
| Yes | | | | | | 45 (45%) | | | | | | | | | | | | 2 (2%) | | | | | | | | | | | |  |  |  |  |  |  |
| **U.2 - Pain disorder associated with psychological factors and a current general medical condition** | | | | | |  | | | | | |  | | | | | |  | | | | | |  | | | | | | 0.170* | | | | | |
| Yes | | | | | | 7 (7%) | | | | | | | | | | | | 2 (2%) | | | | | | | | | | | |  |  |  |  |  |  |
| **V - Current Conduct Disorder** | | | | | |  | | | | | |  | | | | | |  | | | | | |  | | | | | | *** | | | | | |
| Yes | | | | | | - | | | | | | | | | | | | - | | | | | | | | | | | |  |  |  |  |  |  |
| **W.1 - Current Attention Deficit/Hyperactivity Disorder** | | | | | |  | | | | | |  | | | | | |  | | | | | |  | | | | | | 1.000* | | | | | |
| Yes | | | | | | - | | | | | | | | | | | | 1 (1%) | | | | | | | | | | | |  |  |  |  |  |  |
| **W.2 - Current Attention Deficit/Hyperactivity (adult) Disorder** | | | | | |  | | | | | |  | | | | | |  | | | | | |  | | | | | | 1.000* | | | | | |
| Yes | | | | | | 1 (1%) | | | | | | | | | | | | - | | | | | | | | | | | |  |  |  |  |  |  |
| **X - Adjustment Disorder** | | | | | |  | | | | | |  | | | | | |  | | | | | |  | | | | | | 0.721* | | | | | |
| Yes | | | | | | 5 (5%) | | | | | | | | | | | | 3 (3%) | | | | | | | | | | | |  |  |  |  |  |  |
| **Y - Current Probable Premenstrual Dysphoric Disorder** | | | | | |  | | | | | |  | | | | | |  | | | | | |  | | | | | | 0.060 | | | | | |
| Yes | | | | | | 4 (4%) | | | | | | | | | | | | 11 (11%) | | | | | | | | | | | |  |  |  |  |  |  |
| **Z - Current Mixed Anxiety-Depression Disorder** | | | | | |  | | | | | |  | | | | | |  | | | | | |  | | | | | | <0.001 | | | | | |
| Yes | | | | | | 33 (33%) | | | | | | | | | | | | 9 (9%) | | | | | | | | | | | |  |  |  |  |  |  |

* Fisher’s exact test

*** Test not performed due to null variables
